# Amphiphilic Block Copolymer Micelles in Selective Solvents: The Effect of Solvent Selectivity on Micelle Formation

**DOI:** 10.3390/polym11111882

**Published:** 2019-11-14

**Authors:** Labeesh Kumar, Andriy Horechyy, Eva Bittrich, Bhanu Nandan, Petra Uhlmann, Andreas Fery

**Affiliations:** 1 Leibniz-Institut für Polymerforschung Dresden e. V., Hohe Str. 6, 01069 Dresden, Germany; kumar-labeesh@ipfdd.de (L.K.); bittrich-eva@ipfdd.de (E.B.); uhlmannp@ipfdd.de (P.U.); 2 Department of Textile Technology, Indian Institute of Technology Delhi, Hauz Khas, New Delhi 110016, India; nandan@textile.iitd.ac.in; 3 Department of Chemistry, Hamilton Hall, University of Nebraska-Lincoln, 639 North 12th Street, Lincoln, NE 68588, USA; 4 Institute of Physical Chemistry of Polymeric Materials, Technische Universität Dresden, 01062 Dresden, Germany

**Keywords:** block copolymer micelles, selective solvent, solution self-assembly, PS-*b*-P4VP, P4VP corona, spectroscopic ellipsometry, vapor swelling

## Abstract

We investigated the micellar behavior of a series of asymmetric polystyrene-block-poly(4-vinylpyridine) (PS-*b*-P4VP) block copolymers in different P4VP-selective alcoholic solvents. The micellar behavior was further correlated with the spectroscopic ellipsometry results obtained on swelling of PS and P4VP polymer films in the corresponding solvent vapors. The time-resolved (in situ) dynamic light scattering (DLS) measurements, in combination with (ex situ) electron microscopy imaging, revealed information about the aggregation state of PS-*b*-P4VP BCP in different alcohols and the effect of heat treatment. The ellipsometry measurements allowed us to estimate the difference in solvent selectivity toward PS/P4VP pair. Both DLS and ellipsometric studies suggested that less polar alcohols (i.e., 1-propanol, 1-butanol, and 1-pentanol) are likely to be close to each other in terms of their selectivity toward PS/P4VP pair, whereas more polar ethanol and methanol show the highest and the lowest affinity toward P4VP, respectively.

## 1. Introduction

Amphiphilic block copolymers (BCP) attract research interest as template materials for the fabrication of various hybrid nanostructures, such as core–shell, yolk–shell particles, vesicles, porous membranes, or nanofibers, which found their application in various fields [1,2,3,4]. Solution self-assembly provides opportunities to regulate size, shape, morphology, or composition of block copolymer structures and subsequent functional materials [5]. Here, along with the BCP fundamental characteristics, solvent properties, temperature, polymer concentration, or micellization kinetics can be explored to tune the characteristics of the micellar aggregates [6]. 

Block copolymers consisting of poly(vinyl pyridine) segments in their structure are frequently used for the fabrication of various hybrid nanostructures, and polystyrene-block-poly(4-vinylpyridine) (PS-*b*-P4VP) is probably one of the most explored polymers in this area [7]. The ability of pyridine units to undergo hydrogen bonding and coordinate with electron-deficient species largely simplifies the functionalization process and diversifies synthetic strategies, which can be implemented to achieve desired materials. Micellization of symmetric and asymmetric PS-*b*-P4VP BCP was studied in the past by several research groups, focusing on different aspects of micelle formation and morphological transformation processes [8,9,10,11,12,13,14,15]. 

Surprisingly, most of the literature reports related to PS-*b*-P4VP micellar systems have focused on either symmetric or asymmetric BCP compositions with longer PS block where the solution self-assembly was studied in PS-selective conditions. The PS-*b*-P4VP micellar systems comprising longer P4VP blocks are scarce and less explored [12]. Such micelles comprising an inner PS core and outer P4VP corona can be obtained with the aid of P4VP-selective solvents, for instance, alcohols. Alcoholic PS-*b*-P4VP micellar solutions are particularly attractive for the synthesis of various polymer-inorganic core–shell, yolk–shell particles, or hollows particles, by exploring advantages of sol-gel processes [16,17,18]. Although the strong affinity of alcohols toward P4VP is known from the literature [11], to the best of our knowledge, there has been no effort to understand the effect of solvent on the PS-*b*-P4VP micellization process taking place in different alcohols. 

In the present work, we investigated the micellar behavior of PS-*b*-P4VP block copolymers of different *M*_n_ comprising longer P4VP blocks in various alcohols. In particular, we endeavored to understand the effect of solvent and heat treatment on micellar behavior in different alcohols and correlate these results with the strength of solvent selectivity obtained from vapor-swelling experiments on PS and P4VP thin films. 

## 2. Materials and Methods 

Poly(styrene)-block-poly(4-vinylpyridine) (PS-*b*-P4VP) block copolymers of varied molecular weight, as well as polystyrene (PS) and poly(4-vinylpyridine) (P4VP) homopolymers (HP), were purchased from Polymer Source Inc. (Dorval, QC, Canada) and used as received. Main characteristics of BCPs and HPs are summarized in Table 1. Chloroform, toluene and methanol (Fisher Scientific GmbH, Schwerte, Germany), absolute ethanol and 1-butanol (VWR International GmbH, Dresden, Germany), 1-propanol and 1-pentanol (Sigma-Aldrich Chemie GmbH, Taufkirchen, Germany), ammonium hydroxide solution (28%) (Fisher Scientific GmbH, Schwerte, Germany), and hydrogen peroxide (30%) (Merck, Darmstadt, Germany) were of analytical grade and used as received. Highly polished single-crystal silicon wafers ((100) orientation) with ca. 1.5 nm thick silicon oxide layer were purchased from Semiconductor Processing Co. and used as substrates for thin film preparation.

*Dynamic light scattering (DLS).* DLS experiments were carried out using Zetasizer Nano S (ZEN 1600, NIBS Technology, Malvern Instruments, Malvern, UK) equipped with 4 mW He-Ne-laser (632.8 nm, scattering angle 173°). The PS-*b*-P4VP block copolymer was added in the solvent at a concentration of 0.2 mg/mL and stirred at room temperature for at least 70 h, to equilibrate the sample and obtain reproducible DLS results. The quartz cuvette containing 1 mL of BCP solution was placed in Z-sizer, and continuous DLS measurements were started immediately after temperature equilibration (60 s equilibration time). The interval between successive measurements was 2 min. For each measurement point, ten autocorrelation functions (10 s of data collection time per scan) were averaged and evaluated, using Dispersion Technology Software (DTS) appendant to Zetasizer Nano S. DTS includes cumulants analysis and multimodal size-distribution algorithm NNLS, which have been used for the calculation of hydrodynamic particle size and polydispersity index (PDI). The refractive index (RI) of polystyrene latex (n = 1.590) was used as material RI, whereas temperature-dependent values of solvent viscosity were determined form data available in the literature [20].

*Swelling experiments and ellipsometry measurements.* Silicon wafers were precleaned by repeated sonication in dichloromethane, followed by stirring in a mixture of Milli-Q^®^ water, H_2_O_2_, and NH_4_OH (4:1:1 *v*/*v*) for 1 h, at 80 °C. Wafers were thoroughly rinsed with Milli-Q^®^ water and dried with nitrogen flow before being used as substrates for thin film deposition. PS and P4VP polymer films were deposited onto precleaned silicon wafers by spin coating from corresponding polymer solutions in chloroform. Film thickness was adjusted by tuning polymer concentration and rotation speed during spin coating. Time-resolved spectroscopic ellipsometry measurements were carried out at room temperature (23 ± 1 °C), in reflection mode, using a rotating compensator alpha-SE^®^ spectroscopic ellipsometer (J.A. Woollam, Co. Inc., Linkoln, NE, USA). Before measurement of the swelling of polymer films in solvent vapor, optical constants of the specific solvent vapor in equilibrium were analyzed, using a bare Si wafer with a thermally grown SiO_2_ layer of 30 nm. Hence, a silicon wafer with a thermally grown SiO_2_ layer of 30 nm was placed in a quartz cuvette (fixed angle of incidence of 70°, TSL Spectrosil, Hellma, Muellheim, Germany), together with a small aluminum vessel containing 80 µL of solvent. The cuvette was closed with a weighted glass slide, and ellipsometric data were recorded with a time interval of ca. 0.2 min, until equilibrium conditions were reached. For fitting the optical dispersion of the solvent vapor, a model of silicon/SiO_2_ (optical dispersion of Si and SiO_2_ taken from the database), solvent adlayer (fixed Cauchy dispersion), and solvent vapor as ambient (Cauchy dispersion with A and B fitted) were used. The optical constants of the solvent in the liquid phase were measured with refractometry, at a digital multiple wavelength refractometer (DSR-L, Schmidt + Haensch GmbH & Co., Berlin, Germany). The ellipsometric investigations of polymer film swelling were performed in a similar way. Silicon wafers coated with PS or P4VP layer, with a thickness of ca. 50 nm, were placed in the quartz cuvette, along with 80 µL of solvent. The cuvette was closed with a glass slide, and ellipsometric data were recorded until stable plateau values of the film thickness were obtained. Experimental data were fitted with a Cauchy model for the swollen polymer film, using optical constants for solvent vapors at equilibrium. The swelling ratio (*Q*) was calculated by dividing polymer film thickness in the swollen state to the initial film thickness in a dry state. The maximal swelling ratio (*Q*_max_) was calculated by averaging *Q* values at equilibrium over the time interval of ca. 5 min.

*Scanning electron microscopy (SEM).* SEM images were obtained with a Carl Zeiss ULTRA 55 scanning electron microscope (Carl Zeiss SMT, Jena, Germany) at 3 kV acceleration voltage, using an in-lens secondary electron (SE) detector. A drop of BCP alcoholic solution was placed onto a precleaned silicon wafer, and solvent was allowed to evaporate at ambient conditions. All samples were analyzed without any additional coating.

## 3. Results

### 3.1. Micellization of PS-b-P4VP in Alcohol Solutions

For these studies, we used PS-*b*-P4VP(59) block copolymer, with a shorter PS block (φ(PS) ≈ 0.33), which self-assembles into cylindrical morphology in bulk (PS cylinders in P4VP matrix). Upon swelling in P4VP-selective solvents, such as ethanol or methanol, the P4VP matrix can be disintegrated to form isolated nanofibers comprising collapsed PS core and solvent-swollen P4VP corona [21]. Subsequent heat treatment of ethanol dispersion led to the transformation of nanofibers into uniformly sized spherical micelles comprising a collapsed PS core and a solvent swollen P4VP corona. While our previous reports dealt with functionalization of PS-b-P4VP nanofibers and spherical micelles, the present work is addressed to a fundamental understanding of micellization process taking place upon dispersion of PS-b-P4VP BCPs in P4VP-selective solvents, and the role of temperature and solvent selectivity on micelle formation and their characteristics. Alcohols are generally considered to be P4VP-selective solvents for PS/P4VP pair, which are governed by their polar nature and potential for interacting with P4VP units via hydrogen bonding [7,11,12]. The only example on solution assembly of PS-*b*-P4VP containing longer P4VP block, which we found in literature, deals with toluene/ethanol mixtures as an environment [12].

We start our discussion with the PS-*b*-P4VP(59)/ethanol system. The experimental procedure that was used for the preparation of BCP micelles implies direct dispersion (or dissolution) of PS-*b*-P4VP in alcohol and a subsequent equilibration step by stirring the mixture for ca. 70 h, at room temperature. An equilibration step was required to obtain reproducible DLS results and was kept the same for all PS-b-P4VP(59)/alcohol systems. Hence, PS-*b*-P4VP(59)/alcohol solutions were analyzed by DLS before, during, and after heat treatment. 

**Figure 1:**
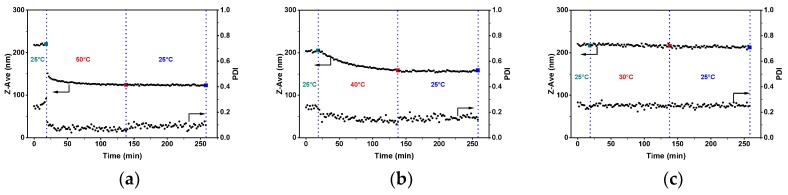
Time-resolved changes of hydrodynamic particle size, Z-ave, and PDI measured by DLS for PS-b-P4VP(59)/ethanol solutions acquired first at 25 °C, upon heat treatment and upon subsequent cooling step. Heat treatments were performed at 50 (**a**), 40 (**b**) and 30 °C (**c**). Vertical dashed lines show time intervals of heating and cooling cycles.

Figure 1 shows DLS data on hydrodynamic size, Z-ave, and PDI of PS-b-P4VP(59) aggregates present in ethanol solutions as a function of time during continuous heating and subsequent cooling steps. At the starting point (i.e., after equilibration), the average hydrodynamic size and PDI of PS-b-P4VP(59) aggregates was 218.6 ± 1.6 nm and 0.257 ± 0.024, respectively. When the solution was heated to 50 °C, both hydrodynamic size and PDI steeply decreased and, after ca. 60 min, reached a plateau at 124.4 ± 0.6 nm and 0.064 ± 0.012, respectively (Figure 1a). The subsequent cooling step led to minor changes in hydrodynamic size, but the PDI was found to be nearly doubled. The same trends in Z-ave and PDI were also revealed upon heating the PS-b-P4VP(59)/ethanol system to 40 °C, and subsequently cooling to 25 °C (Figure 1b). However, a longer time was required to reach the plateau level at this temperature. Moreover, both hydrodynamic size and PDI after heating to 40 °C were larger as compared to those obtained at 50 °C. Finally, no significant changes in size and PDI were observed upon the heating of the PS-b-P4VP(59)/ethanol mixture to 30 °C and subsequently cooling it to 25 °C (Figure 1c).

**Figure 2:**
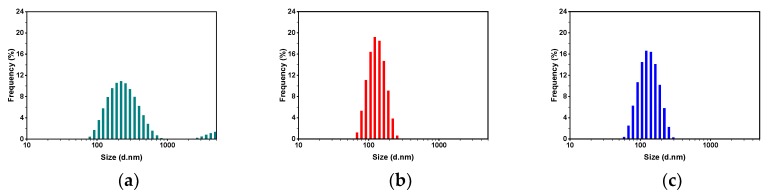

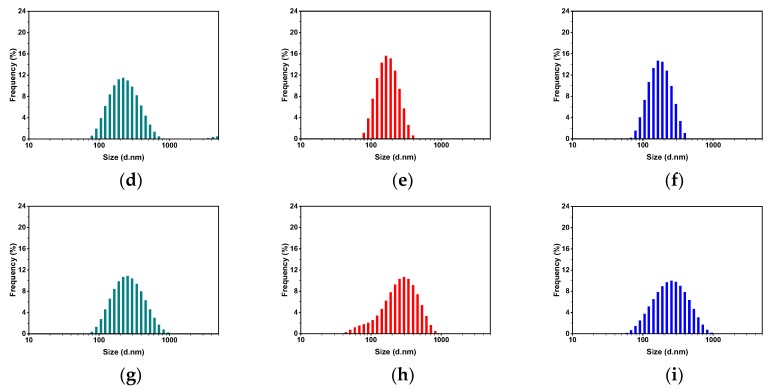
Particle-size-distribution histograms (by intensity) for PS-b-P4VP(59)/ethanol solutions at the last measurement points of different temperature-treatment conditions: (**a**,**d**,**g**) at 25 °C before heating cycle; (**b**,**e**,**h**) upon heating cycle at (**b**) 50, (**e**) 40, and (**h**) 30 °C; and (**c**,**f**,**i**) at 25 °C after heating and subsequent cooling cycles. The corresponding measurement points, which related to each size-distribution histogram, are marked with corresponding colors in Figure 1.

Figure 2 shows size-distribution histograms of PS-b-P4VP(59) aggregates present in ethanol before and after heat treatment at different temperatures. The effect of heat treatment at different temperature regimes on the changes in size distribution (i.e., peak broadness) and maxima positions is clearly seen. The narrowing of size distribution upon heating at higher temperatures was expected, due to the temperature-dependent nature of polymers–solvent interaction (see also Discussion).

**Figure 3:**
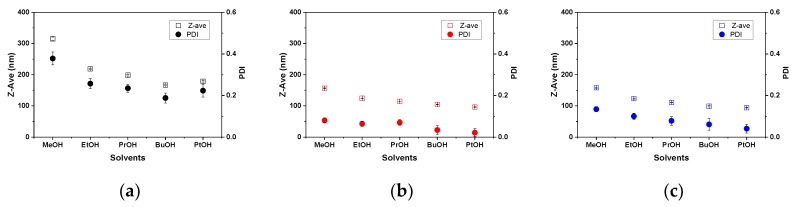
Hydrodynamic particle size, Z-ave, and PDI of PS-b-P4VP(59) aggregates present in different alcohols extracted from plateau regions (**a**) at 25 °C before heating, (**b**) after heating at 50 °C for two hours, and (**c**) after subsequent cooling to 25 °C. Error bars indicate standard deviation determined by averaging DLS data over the last ten measurement points at each temperature regime.

**Figure A1:**
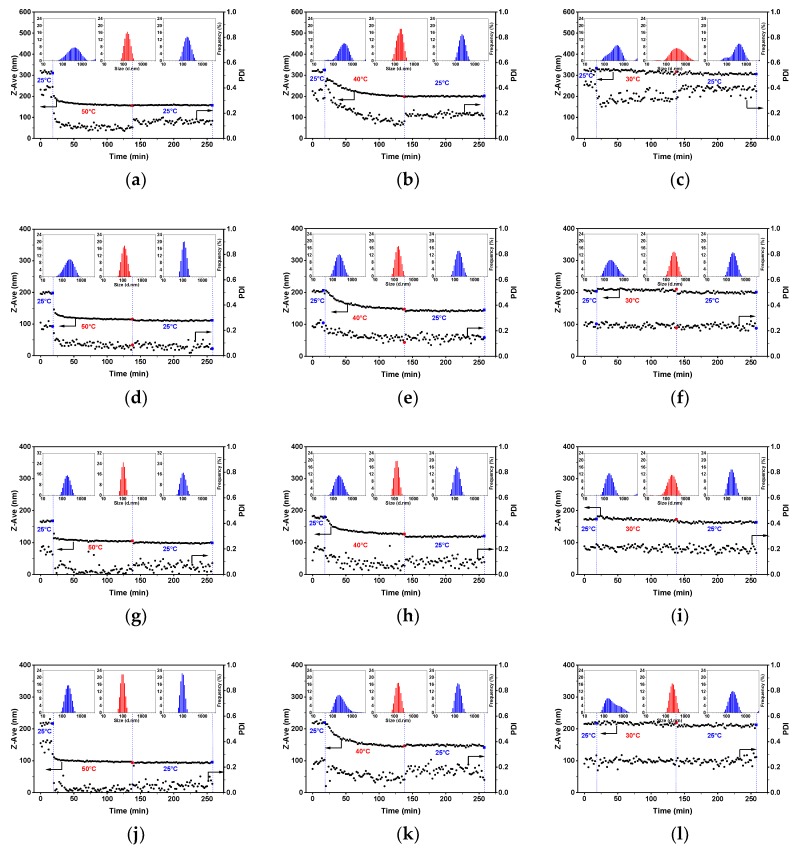
Time-resolved changes of hydrodynamic particle size, Z-ave, and PDI measured by DLS for (**a**–**c**) PS-b-P4VP/methanol, (**d**–**f**) PS-b-P4VP/1-propanol, (**g**–**i**) PS-b-P4VP/1-butanol, and (**j**–**l**) PS-b-P4VP/1-pentanol, solutions acquired first at 25 °C, upon heat treatment and the subsequent cooling step. Heat treatments were performed at 50 (left column), 40 (middle column), and 30 °C (right column). Insets show particle-size distributions (measured by intensity) at the last measurement points taken at different temperatures which are marked in blue (25 °C) and red (30, 40, or 50 °C). Vertical dashed lines point out time intervals of the heating and cooling cycles.

The effect of heat treatment was also investigated for other PS-b-P4VP(59)/alcohol systems. We expected that general features of PS-b-P4VP behavior in other alcohols would be similar to those observed for the PS-b-P4VP(59)/ethanol system. Indeed, all trends in temperature-induced changes of Z-ave and PDI were also maintained for other PS-b-P4VP(59)/alcohols pairs studied (see Figure A1 in Appendix A). Thus, heating at 50 °C was always accompanied by a steep decrease of hydrodynamic size and reduction of PDI, whereas only minor changes in Z-ave and PDI were noticed upon heating solutions at 30 °C. On the other hand, certain dissimilarities were also found for different PS-*b*-P4VP(59)/alcohol systems. Figure 3 summarizes the Z-ave and PDI obtained for different PS-b-P4VP(59)/alcohol dispersions before heating, upon heating at 50 °C for two hours, and upon the subsequent cooling step to 25 °C, averaged over the last ten measurement points at plateau regions. As can be seen, all graphs show clear trends toward the decrease of hydrodynamic size and PDI as the number of methylene groups in alcohol molecules increases. On the other hand, both Z-ave and PDI values before and after heating/cooling steps vary for different alcohols, showing the highest and the lowest differences for PS-b-P4VP(59)/methanol and PS-b-P4VP(59)/1-pentanol systems, respectively (numerical values are given for comparison in Appendix A, Table A1).

### 3.2. Morphology of PS-b-P4VP(59) Aggregates in Different Alcohols

**Figure 4:**
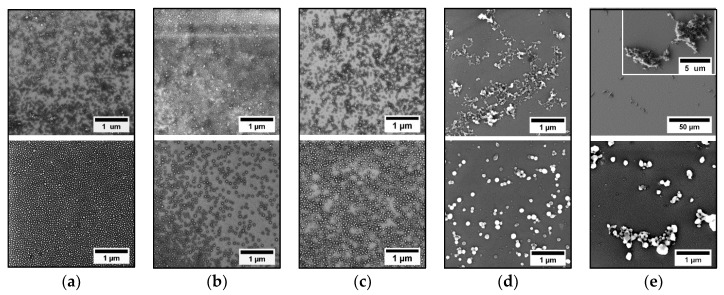
SEM images of PS-b-P4VP(59) assemblies present in different alcohols before heating (upper-line images) and after heating/cooling cycles (lower-line images): (**a**) 1-pentanol; (**b**) 1-butanol; (**c**) 1-propanol); (**d**) ethanol; (**e**) methanol. In all the cases, heat treatment was performed at 50 °C, for 2 h.

PS-*b*-P4VP(59) aggregates present in different PS-b-P4VP(59)/alcohol dispersions were further analyzed in a dry state by SEM. Figure 4 shows SEM images of PS-*b*-P4VP(59) micellar assemblies prepared by drop-casting from different PS-*b*-P4VP(59)/alcohol dispersions before heating (upper line images) and after heating (50 °C)/cooling steps. As can be seen, in 1-pentanol, 1-butanol, and 1-propanol, PS-*b*-P4VP(59) BCP forms spherical micelles already after dissolution and the subsequent equilibration step. After the heating/cooling cycle, micelles appear uniform in size and shape. SEM results on these three systems also revealed formation of particles with relatively narrow size distribution already after the dissolution of BCP in these alcohols. In contrast, predominantly large irregular aggregates were found in the case of the PS-*b*-P4VP(59)/methanol mixture before the heating step (Figure 4d). Similarly, mostly irregular aggregates, along with a small fraction of isolated spherical micelles, were observed in the case of the PS-*b*-P4VP(59)/ethanol mixture, though the size of these aggregates was much smaller as compared to the methanol case. After the heating and subsequent cooling cycles, these aggregates disintegrated into smaller particles, which, nevertheless, were inhomogeneous in size as compared to those formed in higher *M*_w_ alcohols.

### 3.3. The Effect of PS-b-P4VP Molecular Weight

**Figure 5:**
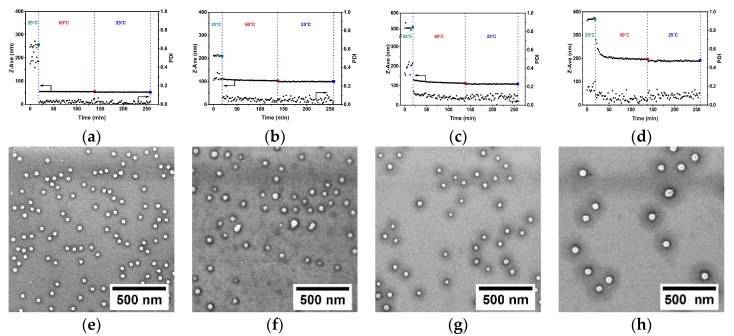
(**a**–**d**) Time-resolved changes of Z-ave and PDI measured by DLS for (**a**) PS-b-P4VP(30)/1-propanol, (**b**) PS-*b*-P4VP(59)/1-propanol, (**c**) PS-*b*-P4VP(75)/1-propanol, and (**d**) PS-b-P4VP(120)/1-propanol solutions acquired first at 25 °C, upon heat treatment at 60 °C, and upon the subsequent cooling step. (**e**–**f**) SEM images of spherical micelles formed upon heating/cooling cycles PS-b-P4VP BCPs of varied *M*_n_ dispersed in 1-propanol: (**e**) PS-b-P4VP(30), (**f**) PS-b-P4VP(59), (**g**) PS-b-P4VP(75), and (**h**) PS-b-P4VP(120).

We also investigated the solution behavior of four different PS-*b*-P4VP BCPs of varied molecular weights (see Table 1 in Experimental Section) during subsequent heating/cooling cycles. DLS experiments were carried out in 1-propanol, maintaining similar experimental conditions as described above. The temperature during the heating cycle was set to 60 °C in order to obtain reproducible DLS data for all PS-b-P4VP/1-propanol systems. After successive heating and cooling steps, PS-b-P4VP/1-propanol micellar solutions were drop-cast onto pre-cleaned silicon wafers and analyzed by SEM. Figure 5 shows Z-ave and PDI plots versus time obtained upon the continuous heating and subsequent cooling steps of four different PS-*b*-P4VP/1-propanol mixtures (numerical values are given for comparison in Appendix A, Table A2), as well as corresponding SEM images of PS-*b*-P4VP micelles present in the respective PS-*b*-P4VP/1-propanol solutions after heating/cooling cycles. As expected, initially present larger and irregular PS-*b*-P4VP aggregates of different PS-*b*-P4VP/1-propanol mixtures disintegrate under heating to form uniform micelles, which is reflected in the decrease of Z-ave and PDI until a stable plateau is reached. As expected, the time intervals required for the formation of uniform micelles increases with an extension of the BCP chain length (compare Figure 5a,d). This could be explained as a result of chain entanglement effects being more pronounced for the longer polymer chains. SEM studies confirmed formation of uniform spherical micelles in all PS-*b*-P4VP/1-propanol mixtures (Figure 5e–h). Notably, when deposited on silicon substrate, PS-*b*-P4VP micelles preserve their core-corona morphology also in dry state, which is a result of the strong affinity of P4VP toward SiO_2_ surface [22].

### 3.4. Swelling of Polymer Films in Solvent Vapors and Ellipsometry experiments

The swelling behavior of PS and P4VP homopolymer thin films, in vapors of different solvents, was investigated using in situ ellipsometry. The swelling behavior was expected to reveal the differences in affinity (or selectivity) of different solvents with respect to each of the BCP components. Swelling of thin polymer films in solvent vapor has indeed been used for the evaluation of solvent affinity and solvent selectivity in the past [23,24]. The advantages of spectroscopic ellipsometry, which affords data over a relatively large sample area and allows determination of the film thickness, along with optical constants of the film layer, were utilized in this study. PS and P4VP polymers were spin-cast onto precleaned silicon wafers as thin films, maintaining the thickness of the film similar for both polymers (50 ± 2 nm). Subsequently, the samples were placed in an in situ ellipsometry cell, together with a small vessel containing 80 µL of solvent, and covered with a glass slide. The swelling process was continued and monitored by ellipsometry, until a maximum in film swelling was reached. At this stage, the equilibrium conditions established were characterized by a plateau in film thickness. The measured film thickness was scaled to the initial film thickness in a dry state, yielding the swelling ratio *Q*(t) at each measurement point, and the maximum swelling ratio, *Q*_max_, at equilibrium. 

**Figure 6:**
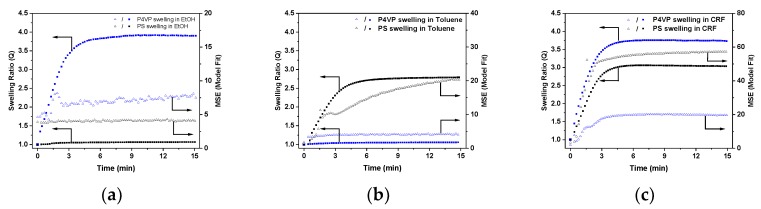
Swelling ratios, *Q*, (filled squares) and MSE values (open triangles) as a function of time upon exposure of PS and P4VP thin films to (**a**) ethanol, (**b**) toluene, and (**c**) chloroform vapors obtained from spectroscopic ellipsometry experiments.

Figure 6 shows the dependency of swelling ratios, *Q*, versus time for PS and P4VP thin films exposed to ethanol, toluene, and chloroform vapors, as obtained from spectroscopic ellipsometry experiments. Mean squared errors (MSE) from the fit model are also given for comparison. The starting point (*t* = 0) corresponds to the dry film state (*Q* = 1), which is the time point when the ellipsometry cell with the sample and solvent was closed with a glass slide. Thus, upon exposure to ethanol vapor, the P4VP film starts to swell immediately after closing the cell and reaches maximum swelling after ca. 10 min of exposure (Figure 6a). Swelling of P4VP was accompanied by an increase in the error of ellipsometry fit (MSE). This could point to a refractive index gradient within the polymer film due to inhomogeneous solvent concentration through the film. This hypothesis is plausibly seen at the early stage of the swelling process, wherein the MSE level first increases and then drops back, probably due to equilibration of solvent concentration in the film. The PS film also swells, but to a very minor extent. An increase of the polymer film thickness at the maximum was almost 50 times higher for P4VP as compared to PS (293% versus 6%), indicating strong selectivity of ethanol toward P4VP. When toluene was used instead of ethanol, the PS swelling ratio was higher as compared to P4VP, corroborating the PS-selective nature of this solvent (Figure 6b). Finally, when polymer films were exposed to the chloroform vapors, both PS and P4VP thin films underwent intensive swelling (Figure 6c). Notably, the maximum swelling of P4VP in chloroform vapors was ca. 20% larger as compared to PS swelling.

**Figure 7:**
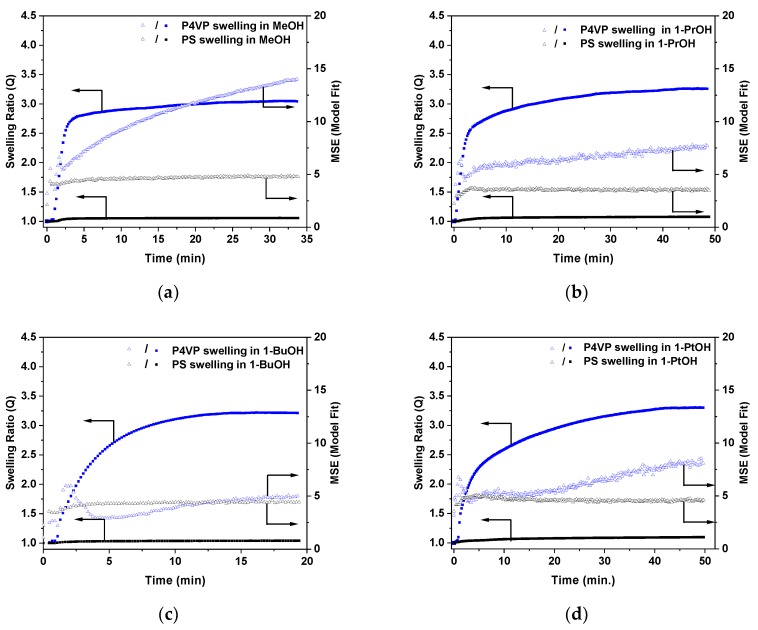
Swelling ratios (*Q*, filled squares) and MSE values (open triangles) as function of time upon exposure of PS and P4VP thin films to (**a**) methanol, (**b**) 1-propanol, (**c**) 1-butanol, and (**d**) 1-pentanol vapors obtained from spectroscopic ellipsometry experiments.

Similar swelling experiments were performed by exposing PS and P4VP thin films to the vapors of other alcohols, and the corresponding plots of swelling ratios, *Q*, versus time are shown in Figure 7. In all alcohols, *Q*_max_ of P4VP was 20 to 50 times higher as compared to PS, corroborating the P4VP-selective nature of these solvents. However, as is discussed further on in the paper, vapor-swelling experiments also revealed certain dissimilarities in selectivity of different alcohols toward the PS/P4VP pair.

## 4. Discussion

We first discuss our results on vapor-swelling experiments in more details. The affinity of a given solvent toward a particular polymer can be estimated from the difference in solubility parameters of the components:

(1) $$\chi_{P,S}=\frac{V_{S}}{RT}{\left(\delta_{S}-\delta_{P}\right)}^{2}+0.34$$

where $\chi_{P,S}$ is the polymer–solvent interaction parameter, $V_{S}$ is the molar volume of the solvent, *R* is gas constant, *T* is the absolute temperature, and $\delta_{S}$ and $\delta_{S}$ are the solubility parameters of solvent and polymer, respectively [23]. The first term in Equation (1) represents enthalpic contributions, whereas the second term is a correction factor for the entropic contributions. The inverse relationship between $\chi_{P,S}$ and *T* explains well the effect of different temperatures on micellar size. This estimation method, however, is limited to particular polymer–solvent systems, since it does not account for polar- and hydrogen-bonding interactions between the components. Moreover, even for well-studied polymers, such as polystyrene, the solubility parameter values vary from each other [7,9,24]. Alternatively, the relative energy difference (RED) concept has been used to evaluate the polymer–solvent interactions and accounts for polar- and hydrogen-bonding interactions:

(2) $$R_{a}^{2}=4*{\left(\delta_{d,S}-\delta_{d,P}\right)}^{2}+{\left(\delta_{p,S}-\delta_{p,P}\right)}^{2}+{\left(\delta_{h,S}-\delta_{h,P}\right)}^{2}\;\;$$

$$RED=R_{a}/R_{0}$$

where $\delta_{d,P}$, $\delta_{p,P}$, $\delta_{h,P}$, and $\delta_{d,S}$, $\delta_{p,S}$, $\delta_{h,S}$ are the Hansen solubility parameters (HSP) of the polymer and the solvent, which represent dispersive, polar, and hydrogen bonding components, respectively, whereas $R_{0}$ is the radius of the sphere enclosing good solvents [25]. Though the RED approach can be implemented to the polar systems, its application is limited to the polymers with known HSP and $R_{0}$ values. While for PS these data are available, we could find only one report providing HSP values for P4VP (see Table A4 in Appendix A) [14]. Unfortunately, we were not able to track back the original source for these values. We also could not find *R*_0_ value for P4VP, which restricts the possibility to implement RED method to any of P4VP/solvent systems. Most significant, both $\chi_{P,S}$ and *RED* (or $R_{a}$) values based on the available solubility parameter values do not match well with the experimental observation on particular polymer–solvent systems (see Table A5 and the following discussion in Appendix A). Therefore, swelling experiments on thin PS and P4VP films exposed to the vapors of different solvents were carried out to revel the differences in their affinity. 

**Figure 8:**
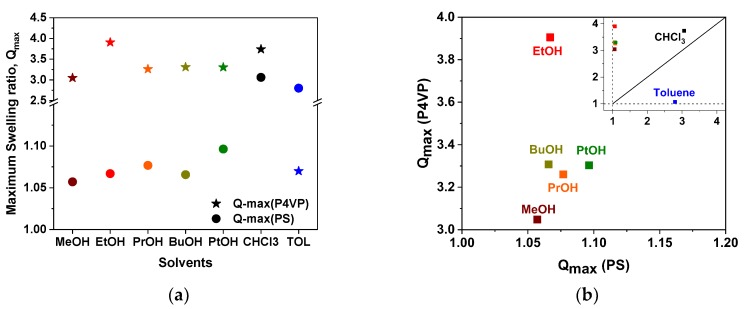
(**a**) Maximum swelling ratios, *Q*_max_, of PS (circles) and P4VP (stars) thin films exposed to the vapors of different solvents determined by spectroscopic ellipsometry. (**b**) *Q*_max_(P4VP) versus *Q*_max_(PS) plot depicting the location of alcohols in the P4VP selective region. The inset on (**b**) is the same plot, which covers both PS- and P4VP-selective regions and includes a “solvent neutrality line” (see text below for more details).

Figure 8a summarizes the averaged values of *Q*_max_ obtained upon swelling of PS and P4VP in alcohol vapors, as well as in vapors of toluene and chloroform. As expected, in alcohol vapors, P4VP swells more than PS and *Q*_max_(P4VP) ranges from 3 to 4 for different alcohols, while *Q*_max_(PS) varies from 1.05 to 1.10 only. The situation turns opposite in toluene vapors, where the PS swelling dominates over P4VP. Since low *M*_w_ alcohols and toluene are known as P4VP- and PS-selective solvents, respectively, these results are in full agreement with our expectations. Chloroform can solubilize both PS and P4VP, being often considered as a good solvent for both blocks. Thus, in chloroform vapors, swelling of both P4VP and PS was comparatively high, though P4VP swelling was higher as compared to PS. 

We assume that after reaching a plateau level in the polymer swelling ratio, an equilibrium is maintained between solvents in the vapor phase, in the liquid phase, and in the polymer layer, and no significant solvent leakage out of the cell took place with time. In fact, the measured film thickness was stable within a time interval over multiple measurement points, suggesting that the solvent concentration in the vapor phase was constant and the equilibrium conditions were maintained. However, a direct comparison of the swelling results in vapors of different solvents is less realistic due to the difference in their vapor pressure (see Table A3 in Appendix A). Nevertheless, the comparison is valid for different polymers swollen in the same solvent if the same experimental conditions are maintained for both the polymers. Thus, we plotted *Q*_max_ results as *Q*_max_(P4VP) versus *Q*_max_(PS), which are shown in Figure 8b [26]. The results on *Q*_max_ obtained from swelling of PS and P4VP in toluene and chloroform vapors are included for comparison (Figure 8b, inset). The “solvent neutrality line” defined by *Q*_max_(P4VP) = *Q*_max_(PS), which splits the graph into the P4VP-selective and PS-selective regions, is also shown. As expected, in the *Q*_max_–*Q*_max_ plot, toluene locates in the PS-selective region, i.e., strong PS swelling and minor P4VP swelling. Next, the position of chloroform, which is known as a good solvent for both PS and P4VP [11], is close to the neutral line (Figure 8b, inset), though its affinities toward PS and P4VP seem to be slightly different. Finally, all alcohols are located in the P4VP-selective region and show rather minor differences in position along the *Q*_max_(PS) axis. Interestingly, three higher *M*_w_ alcohols, 1-propanol, 1-butanol, and 1-pentanol, are located close to each other also along the *Q*_max_(P4VP) axis. Considering that, in these solvents, PS-b-P4VP demonstrated distinct similarity also in solution phase, we speculate that, at specified conditions, these three alcohols should be very close to each other in terms of their selectivity for PS/P4VP pair. Ethanol stands separately and shifts toward the P4VP-selective region, which must be the consequence of the greater polarity and higher vapor pressure of this solvent. In contrast, methanol is shifted toward smaller *Q*_max_(P4VP), i.e., lower affinity toward P4VP. Considering that saturated vapor pressure of methanol at 25 °C is more than twice that of ethanol, the very high polarity of methanol might be the reason for the reduced affinity toward P4VP, as compared to other alcohols (see Table A3 in Appendix A). 

Coming back to the results on the solution behavior of PS-*b*-P4VP in different alcohols, several aspects should be addressed in view of solvent vapor-swelling experiments. For a given BCP, which forms spherical micelles in selective solvents, there are two factors which define the hydrodynamic micellar size at specified experimental conditions. The first factor is the aggregation number (N_agg_), which depends on the polymer/solvent Flory–Huggins interaction parameter, χ_P,S_ [27]. The second factor is the conformation of polymer chains comprising the core and shell of the micelle, which also depends on the strength of interaction of each block with a particular solvent. In selective solvents, which are good for P4VP and poor for PS, like alcohols, PS-*b*-P4VP micelles comprise collapsed PS core and swollen P4VP corona. As the affinity of the solvent toward the corona-forming block is reduced, chains will adopt less stretched conformation, which will lead to the lowering of hydrodynamic micelle size. On the other hand, if the solvent quality for the core-forming block increases, the micellar core will swell due to additional chain stretching and an increase of solvent fraction accommodated in the micellar core. This is valid for the range of selective solvents (or solvent mixtures) in which micelles are stable and do not disintegrate, as the solvent selectivity changes. The effect of solvent selectivity on N_agg_ and micellar size was reported by Park and co-workers for symmetric PS-*b*-P4VP, in a series of ethanol/toluene mixtures with different solvent ratios [11]. They showed that the hydrodynamic size, the core size, and the aggregation number of spherical PS-*b*-P4VP micelles gradually decreased upon lowering the selectivity of the solvent mixture. The hard and large spherical micelles were formed at highly selective solvent mixtures, while the soft and small micelles were formed at less-selective solvent mixtures. Due to the asymmetric composition of PS-*b*-P4VP BCP comprising longer P4VP block and preferential solvation of P4VP block by alcohols, we expected formation of star-like (or core-corona) micelles in these solvents. These micellar structures should adopt core–shell morphology upon drying. Indeed, the core–shell morphology of micellar structures present in higher *M*_w_ alcohols can be clearly seen on the corresponding SEM images (Figure 3a–c and Figure 5e–h). The central part of the micelle appears brighter, which is due to the topographical contrast provided by the PS core. The outer part of the micelle appears darker due to the elemental contrast, which originates from a thin P4VP layer adhered to the silicon substrate and surrounding the micellar core. The situation, however, turns different in PS-*b*-P4VP/ethanol and PS-b-P4VP/methanol systems. In both cases, relatively large and broadly distributed spherical particles, which, plausibly, belong to so-called large compound micelles, are predominantly visible, along with a minor fraction of star-like micelles. Moreover, the in PS-*b*-P4VP/methanol system, there are still larger and irregularly shaped particles, which are aggregates or clusters of several individual particles. Nevertheless, the above results revealed for different PS-*b*-P4VP/alcohol systems correlate with results obtained by Park and co-workers for symmetric PS-b-P4VP in strongly selective ethanol/toluene mixtures [11]. For comparison, star-like micelles comprising P4VP core and PS corona were also found in the case of symmetric and PS-longer PS-*b*-P4VP BCPs in PS-selective solvents, such as toluene or THF [8,11], while for PS-*b*-P4VP with a longer P4VP block, vesicular aggregates were found instead [12]. It should be pointed out that, by using the same PS-*b*-P4VP BCP, smaller and uniform in size star-like micelles can be also obtained in ethanol and methanol if the block polymer is first solubilized in a common good solvent and then transferred to a P4VP-selective alcohol [18,21]. The above difference can be explained as follows. The molecular exchange between individual micelles in case of BCPs is very slow as compared to a classical surfactant micelle. If the solvent cannot efficiently solvate one of the blocks, this block will remain in the frozen (glassy) state, though the second block is well soluble. If the polymer is directly added to a highly selective solvent, i.e., without pre-dissolution in a good solvent, the initial aggregates will be preserved and additional heat treatment is required to disintegrate them. This situation is seen in the case of PS-*b*-P4VP/methanol system, where the large aggregates dominate before the heating step. These observations are consistent with ellipsometry results for polymer swelling in methanol vapors. Despite the highest vapor pressure, the ability of methanol to swell PS and P4VP was the lowest among all alcohols studied. In contrast, dissolution of PS-b-P4VP in 1-propanol, 1-butanol, and 1-pentanol under stirring permits disintegration of aggregates and formation of spherical micelles already at RT. This clearly indicates that these solvents can better solvate PS block as compared to more polar methanol and ethanol, which is also in agreement with our expectation. Vapor swelling results also suggest that these three alcohols might be very close to each other in terms of their selectivity toward PS/P4VP pair. 

**Figure 9:**
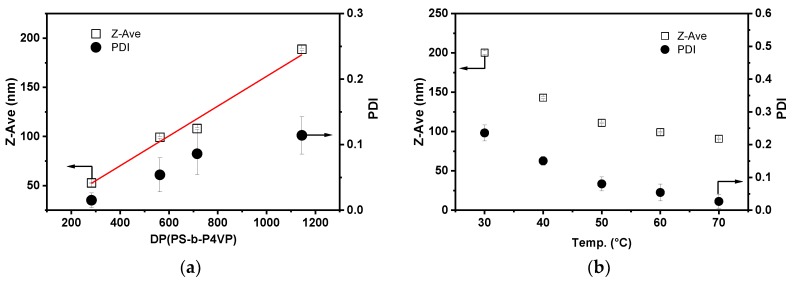
(**a**) Z-ave and PDI of PS-b-P4VP micelles present in different PS-b-P4VP/1-propanol mixtures as function of PS-b-P4VP degree of polymerization obtained after heating at 60 °C and subsequent cooling to 25 °C. Straight line represents the linear fit of experimental data; (**b**) hydrodynamic particle size, Z-ave, and PDI of PS-b-P4VP assemblies present in PS-b-P4VP(59)/1-propanol mixtures obtained after heating steps performed at different temperatures and subsequent cooling to 25 °C.

The effect of BCP chain length on PS-b-P4VP micellar size and the effect of temperature are summarized in Figure 9. Expectedly, with an increase of the BCP chain length, the hydrodynamic size of PS-b-P4VP micelles increases (Figure 9a). Moreover, the hydrodynamic micellar size shows linear dependency versus the overall degree of polymerization (DP) of BCP, which is in accordance with scaling relation derived for the amphiphilic BCP micelles in selective solvents [9]. The effect of temperature on hydrodynamic micellar size is shown in Figure 9b. As can be seen, after successive heating/cooling cycles maintained at different temperatures, the final hydrodynamic micellar size gradually decreases and reaches the minimum values at the highest temperature. Performing experiments at higher temperatures was hardly possible because of enhanced solvent-evaporation effects. Considering the trend in the change of hydrodynamic particle size, one may expect that the further increase of temperature would lead to a plateau level where no further changes in micellar size will happen. However, this may not be the case because of following reasons. There are two processes which contribute to the changes in hydrodynamic size of BCP assemblies upon heating. The first process is the disintegration of PS-*b*-P4VP aggregates, which is characterized by substantial decrease of Z-ave and PDI. The second process is the reduction of the aggregation number of BCP micelles upon heating, which also results in a decrease in micellar size. The latter is associated with temperature-dependent changes in the polymer–solvent interaction parameter. Heat treatment provides more favorable condition for the solvation of core-forming block (PS) at elevated temperatures. This will ultimately lead to a reduced number of polymer chains (i.e., PS blocks) which could be confined within the micellar core. 

## 5. Conclusions

In summary, we investigated the effects of solvent nature, block copolymer molecular weight, and temperature on solution behavior of asymmetric PS-b-P4VP block copolymers in a series of alcohols as P4VP-selective solvents. The results were compared with the spectroscopic ellipsometric results obtained on swelling of PS and P4VP thin films exposed to the solvent vapors. Time-resolved DLS experiments, in combination with electron microscopy studies, reveal that, in higher *M*_w_ alcohols (1-propanol, 1-butanol, and 1-pentanol), spherical micelles comprising collapsed PS core and swollen P4VPcorona can be obtained upon direct dissolution of PS-*b*-P4VP in these solvents. Subsequent heat treatment further leads to the formation of spherically shaped uniform micelles with relatively low PDI. The size of PS-b-P4VP micelles in these three solvents decreases with an increase of aliphatic chain length in alcohol molecules, plausibly due to the decrease of the micellar aggregation number. In contrast, mostly irregular aggregates were present in more polar methanol and ethanol under similar experimental conditions, plausibly, due to the very limited solvation of PS block in these polar solvents. Spectroscopic ellipsometry studies on PS and P4VP thin films exposed to the solvent vapors reveal that the higher *M*_w_ alcohols (1-propanol, 1-butanol, and 1-pentanol) are likely to be very close to each other in terms of their affinity toward PS and P4VP. Ethanol shows the highest, and methanol shows the lowest, affinity toward P4VP. The latter might be the consequence of the too-high polarity of methanol, which reduces the solvation effects of this solvent. 

## Figures and Tables

**Table 1:** Characteristics of block copolymers and homopolymers used in present work.

BCP or HP Assignments	*M*_n_(PS) (g mol^−1^)	*M*_n_(P4VP) (g mol^−1^)	*M*_n_(PS-b-P4VP) (g mol^−1^)	PDI	DP	φ(PS) ^b^
PS-b-P4VP(30) ^a^	10,400	19,200	29,600	1.27	282	≈0.36
PS-b-P4VP(59)^a^	18,500	40,500	59,000	1.10	563	≈0.33
PS-b-P4VP(75)^a^	24,000	51,000	75,000	1.10	716	≈0.33
PS-b-P4VP(120)^a^	38,000	82,000	120,000	1.28	1145	≈0.33
PS	51,000	-	-	1.05	490	-
P4VP	-	48,000	-	1.07	457	-

^a^ The numbers in parentheses denote the total *M*_n_ of BCPs; ^b^ φ are the volume fractions of PS block calculated from the block ratios using 1.05 and 1.11 g cm^−3^ as densities of PS and P4VP, respectively [19].

## Appendix A

**Table A1:** Numerical values of hydrodynamic size (Z-ave) and PDI of PS-b-P4VP(59) assemblies in different alcohols before and after heating/cooling cycles.

Solvent	Z-ave ^a^ (nm) at 25 °C	PDI ^a^ at 25 °C	Z-ave ^b^ (nm) at 50 °C	PDI ^b^ at 50 °C	Z-ave ^c^ (nm) at 25 °C	PDI ^c^ at 25 °C
Methanol	315.2 ± 5.6	0.378 ± 0.031	156.9 ± 0.8	0.080 ± 0.012	158.1 ± 1.1	0.134 ± 0.012
Ethanol	218.6 ± 1.6	0.257 ± 0.024	124.4 ± 0.6	0.064 ± 0.012	123.4 ± 1.1	0.100 ± 0.015
1-Propanol	198.4 ± 2.6	0.235 ± 0.020	114.9 ± 0.1	0.070 ± 0.013	111.1 ± 1.0	0.078 ± 0.021
1-Butanol	166.8 ± 2.0	0.188 ± 0.024	104.7 ± 0.7	0.034 ± 0.021	98.7 ± 1.6	0.061 ± 0.028
1-Pentanol	178.9 ± 2.1	0.223 ± 0.031	96.3 ± 0.6	0.021 ± 0.019	94.0 ± 0.7	0.041 ± 0.021

^a^ Measured at 25 °C, before the heating step; ^b^ measured at 50 °C, at the end of the heating cycle; ^c^ measured at 25 °C at the end of cooling cycle. Data represent the average values over the last 10 measurement points at each temperature regime.

**Table A2:** Numerical values of hydrodynamic size (Z-ave) and PDI of PS-b-P4VP assemblies in 1-propanol before and after heating/cooling cycles obtained for BCPs of varied molecular weights.

Block Copolymer	Z-ave ^a^ (nm) at 25 °C	PDI ^a^ at 25 °C	Z-ave ^b^ (nm) at 60 °C	PDI ^b^ at 60 °C	Z-ave ^c^ (nm) at 25 °C	PDI ^c^ at 25 °C
PS-b-P4VP(30) ^a^	253.9 ± 8.2	0.473 ± 0.046	54.6 ± 0.3	0.028 ± 0.017	52.7 ± 0.4	0.015 ± 0.011
PS-b-P4VP(59) ^a^	211.4 ± 2.6	0.299 ± 0.0270	103.2 ± 0.5	0.044 ± 0.015	99.1 ± 0.9	0.054 ± 0.025
PS-b-P4VP(75) ^a^	508.1 ± 12.0	0.405 ± 0.052	112.9 ± 0.6	0.068 ± 0.010	108.1 ± 1.0	0.086 ± 0.031
PS-b-P4VP(120) ^a^	368.6 ± 3.5	0.185 ± 0.036	196.7 ± 1.4	0.062 ± 0.018	188.1 ± 1.2	0.121 ± 0.026

^a^ Measured at 25 °C before the heating step; ^b^ measured at 60 °C, at the end of heating cycle; ^c^ measured at 25 °C at the end of cooling cycle. Data represent the average values over the last ten measurement points at each temperature regime.

**Table A3:** Vapor pressure (VP) at 25 °C [28], molar volume (V_m_), and Hansen solubility parameters (HSP) of the solvents used [25].

Solvent	VP at 25 °C ^a^ (kPa)	V_m_ (cm^3^ mol^−1^)	δ_d_^b^ (J cm^−3^)^1/2^	δ_p_^b^ (J cm^−3^)^1/2^	δ_h_^b^ (J cm^−3^)^1/2^	δ_t_^c^ (J cm^−3^)^1/2^
Methanol	16.7	40.7	15.1	12.3	22.3	29.6
Ethanol	8.7	58.5	15.8	8.8	19.4	26.1
1-Propanol	3.3	74.8	16.0	6.8	17.4	24.4
1-Butanol	1.3	91.5	16.0	5.7	15.8	23.3
1-Pentanol	0.4	108.2	16.0	4.5	13.9	21.6
Toluene	3.9	106.8	18.0	1.4	2.0	18.3
Chloroform	25.5	80.7	17.8	3.1	5.7	19.0
THF	23.5	81.7	16.8	5.7	8.0	19.5

^a^ Solvent vapor pressure was determined using Antoine equation and Antoine coefficients from [28]; ^b^ δ_d_, δ_p_, and δ_h_ are dispersive, polar, hydrogen bonding Hansen solubility parameters, respectively; ^c^ δ_t_ is a total solubility parameter calculated as $\delta_{t}^{2}=\delta_{d}^{2}+\delta_{p}^{2}+\delta_{h}^{2}$.

**Table A4:** Solubility parameters of PS, P2VP, and P4VP reported in literature.

Polymer	δ_H_^a^ (J cm^−3^)^1/2^	δ_d_^b^ (J cm^−3^)^1/2^	δ_p_^b^ (J cm^−3^)^1/2^	δ_h_^2 b^(J cm^−3^)^1/2^	δ_t_^c^ (J cm^−3^)^1/2^	R_0_	Ref.
PS	18.5	-	-	-	-	-	[7]
P2VP	21.3	-	-	-	-	-	[29]
P4VP	24.0	-	-	-	-	-	[30]
PS	-	18.5	4.5	2.9	19.3	8	[25]
P4VP	-	18.1	6.8	7.2	20.6	?	[14]

^a^ δ_H_ is a Hildebrand solubility parameter; ^b^ δ_d_, δ_p_, and δ_h_ are HSP for dispersive, polar, H-bonding components, respectively; ^c^ δ_t_ is a total solubility parameter calculated form $\delta_{t}^{2}=\delta_{d}^{2}+\delta_{p}^{2}+\delta_{h}^{2}$.

**Table A5:** Solvent–polymer interaction parameters, Ra, and RED values calculated for different polymer–solvent pairs.

				PS/Solvent		P4VP/Solvent	
Solvent	χ_S-PS_^a^	χ_S-P2VP_^a^	χ_S-P4VP_^a^	R_a_^b^	RED ^b^	R_a_^b^	RED ^b^
Methanol	2.51	**1.55**	**0.89**	22.0	2.75	**17.2**	?
Ethanol	1.89	**1.00**	0.49	17.9	2.24	**13.2**	?
1-Propanol	1.48	**0.67**	0.35	15.5	1.94	**11.0**	?
1-Butanol	1.14	0.47	0.37	13.9	1.73	**9.6**	?
1-Pentanol	0.77	0.35	0.38	12.1	1.51	**8.3**	?
Toluene	0.34	0.66	1.44	3.4	0.42	**7.5**	?
Chloroform	0.35	**0.58**	**1.45**	3.4	0.43	**4.4**	?
THF	0.37	**1.55**	**0.89**	6.3	0.78	**2.9**	?

^a^ Calculated from Hildebrand solubility parameters given in Table A3, using Equation (1); ^b^ calculated using Hansen solubility parameters given in Table A3, using Equation (2).

The values of *χ_S-P_*, *R_a_*, and *RED* were calculated by using Equations (1) and (2), based on the solubility parameters listed in the Table A3. The entries, which are marked in bold, represent the polymer–solvent pairs, which do not match with the experimental data. For instance, methanol, ethanol, and propanol are known as good solvents for P2VP and P4VP, while the calculated χ values reveal them as poor solvents (i.e., χ > 0.5), which is not the case. Similarly, *χ_S-P2VP_* and *χ_S-P4VP_* for THF and chloroform reveal them as poor solvents. However, chloroform dissolves P2VP and P4VP, while THF is a good solvent for P2VP only. Next, the RED values for P4VP-solvent pairs cannot be calculated because of unknown *R*_0_ for this polymer. However, the calculated *Ra* values indicate that toluene is a better solvent for P4VP as compared to all the alcohols, which is not the case. Similarly, THF should dissolve P4VP better then chloroform, which is also not the case. These examples clearly show the limitations of exploring solubility parameters for evaluation and comparison of polymer–solvent interactions and solvent selectivity, which were recently addressed for other polymer–solvent systems [24].
